# Supplementary material for: Epithelial-mesenchymal plasticity determines estrogen receptor positive breast cancer dormancy and epithelial reconversion drives recurrence
Source: Nat Commun. 2022 Aug 25;13:4975. doi: 10.1038/s41467-022-32523-6 (PMC9411634; doi:10.1038/s41467-022-32523-6)
Supplement: Supplementary file 1 — Supplementary Information [file 41467_2022_32523_MOESM1_ESM.pdf]

Supplementary Information

Epithelial-mesenchymal plasticity determines estrogen receptor positive breast cancer dormancy and epithelial conversion drives recurrence

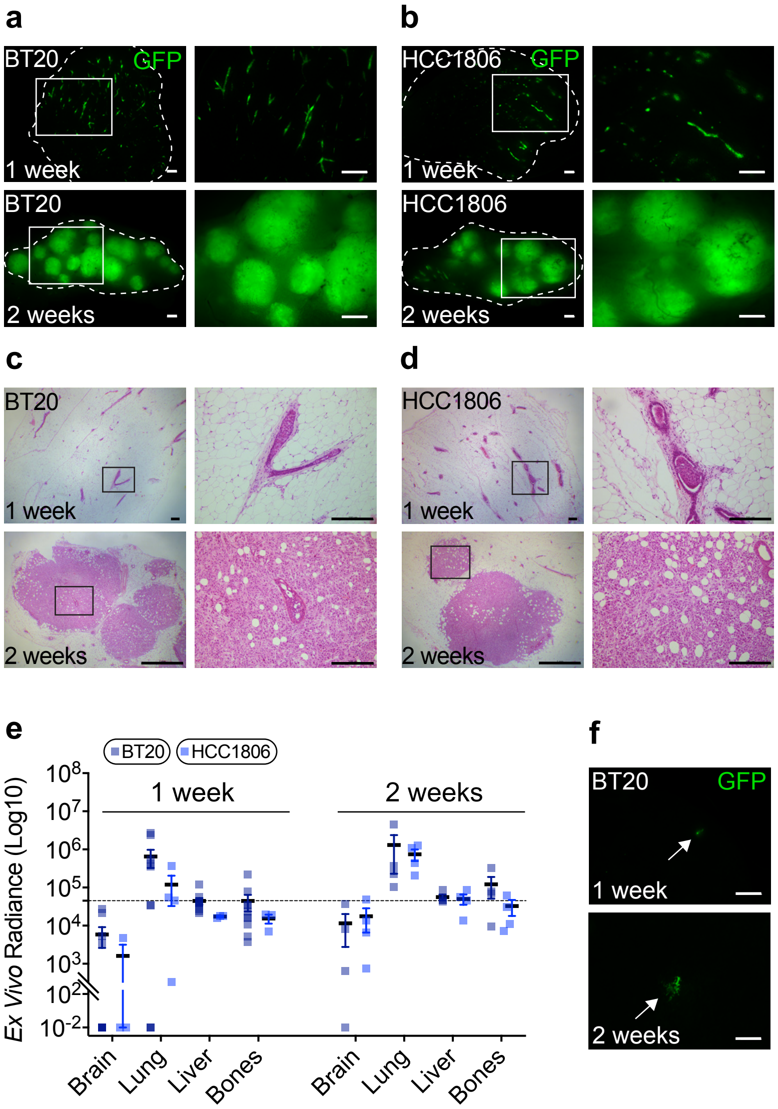

Supplementary Fig. 1. Early dissemination of TN BC cells *in vivo*

a, b. Representative fluorescence stereo micrographs of mammary glands 1 week (top) and 2 weeks (bottom) after intraductal injection with BT20 **(a)** and HCC1806 **(b)**. Scale bars, 1 mm. **c, d.** Representative H&E micrographs of BT20 **(c)** and HCC1806 **(d)** intraductal xenografts 1 week (top) and 2 weeks (bottom) after injection. Scale bars, 1 mm. **e.** *Ex vivo* bioluminescence of organs from mice bearing BT20 and HCC1806 1 week from 10 and 4 mice, respectively, (left) and 2 weeks from 4 mice (right) after intraductal injection. **f.** Representative fluorescence stereo micrographs of lungs from n=2 BT20 intraductal xenografts-bearing mice 1 week (top panel) and 2 weeks (bottom panel) after intraductal injection. The arrow points to TN DTCs. Scale bars, 50 μm.

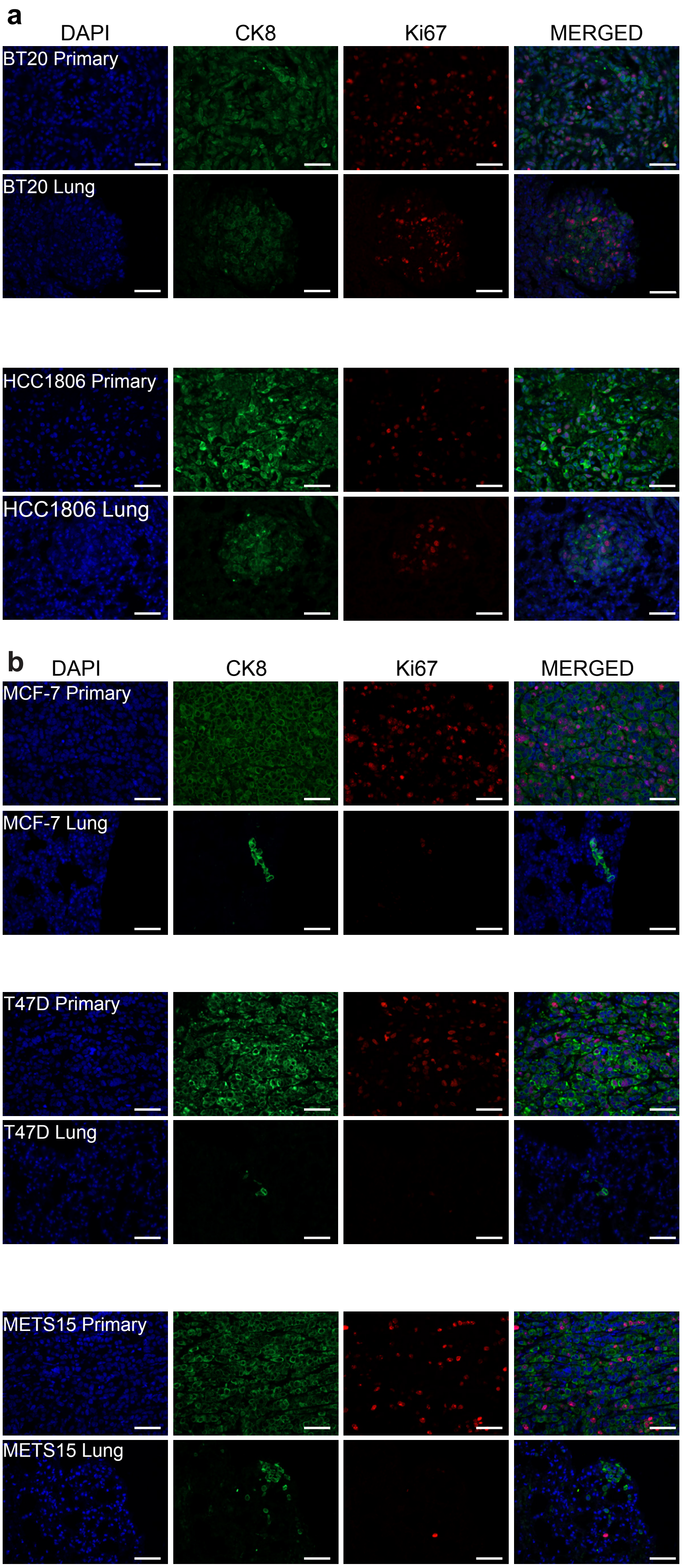

Supplementary Fig. 2. Low proliferative indices of ER⁺ lung DTCs

a, b. Representative IF for CK8 (green) and Ki67 (red), counterstained with DAPI (blue), of matched primary and lung metastases of TN (a) and ER⁺ **(b)** BC intraductal xenografts-bearing mice. Scale bars, 50 μm.

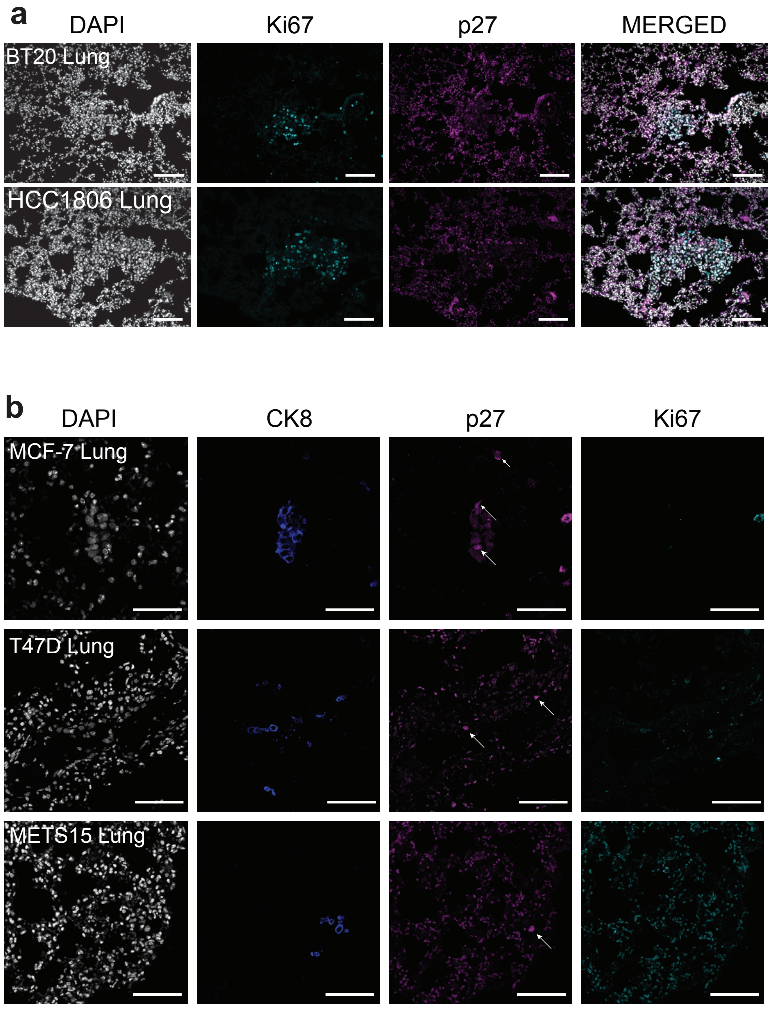

Supplementary Fig. 3. Quiescence marker in ER$^+$ and TN DTCs

a. Representative immunofluorescence micrographs for p27 (magenta) and Ki67 (cyan), counterstained with DAPI (gray), on lung section from mice xenografted with BT20 and HCC1806. **b.** Representative immunofluorescence micrographs for CK8 (blue), p27 (magenta) and Ki67 (cyan), counterstained with DAPI (gray), on lung section from MCF-7$^-$, T47D$^-$, and METS15-bearing mice. Arrows point to p27$^+$/Ki67$^-$ lung DTCs. Scale bar, 50 μm.

Supplementary Figure 4

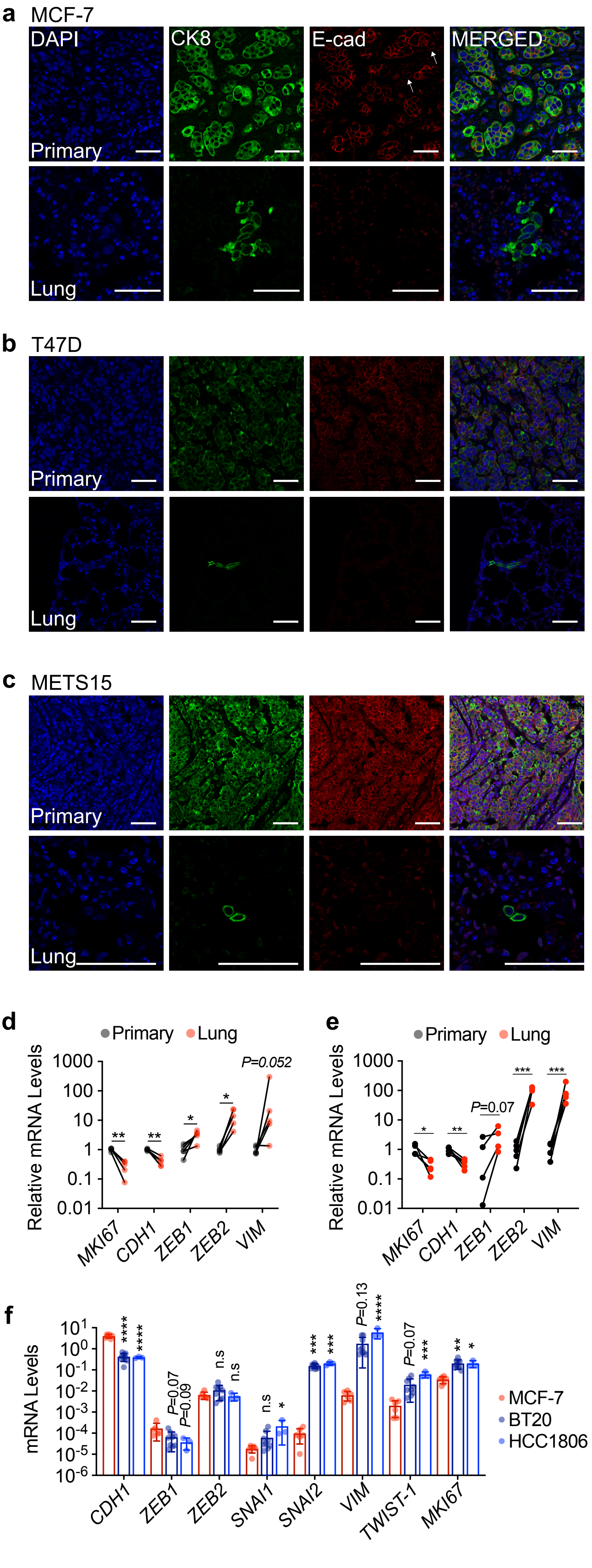

Supplementary Fig. 4. Dormant DTCs from ER+ intraductal xenografts have low E-cad and increased EMT-TFs

a-c. Representative immunofluorescence micrographs stained for CK8 (green) and E-cad (red), counterstained with DAPI (blue), from 3 matched MCF-7 (**a**), T47D (**b**), and METS15 (**c**) primary tumors and lungs. Scale bar, 50 μm. **d, e.** Relative *KI67* and EMT marker mRNA levels normalized to the geometric mean of *GAPDH* and *HPRT* transcript levels in matched primary tumors and micro-dissected lung lesions (**d**) or primary tumor and lung tissue (**e**) from 5 mice intraductally engrafted with METS15 cells. Two-tailed paired t-test. **f.** mRNA levels of EMT related genes in MCF-7 (n=6), BT20 (n=9), and HCC1806 (n=3) intraductal xenografts, respectively, from at least 3 mice. mRNA levels were normalized to the geometric mean of *GAPDH* and *HPRT*. Data represent mean ± SD. One-way ANOVA. *, **, ***, ****, and n.s., represent $P<0.05$, 0.01, 0.001, 0.0001, and not significant, respectively.

Supplementary Figure 5

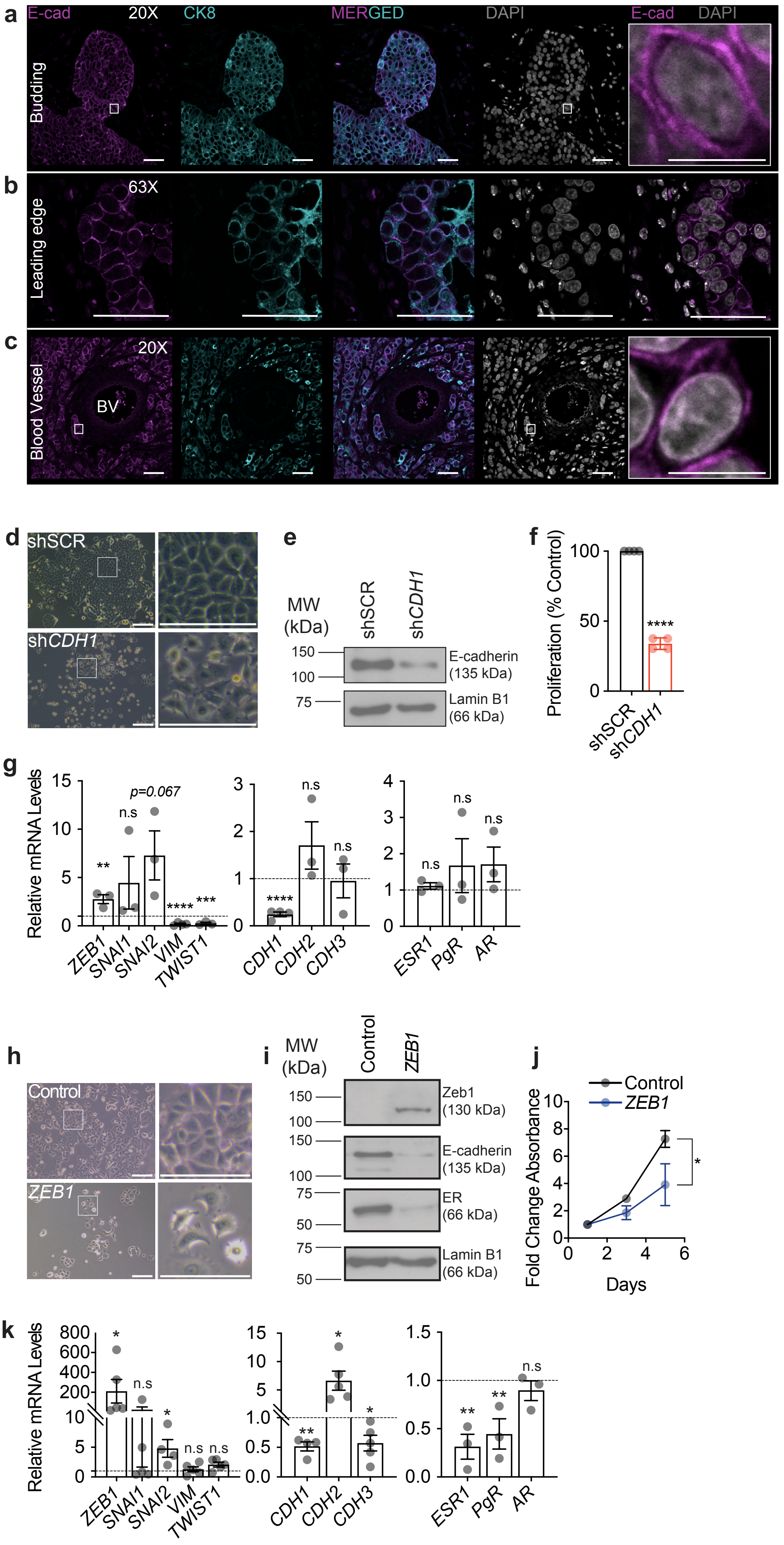

Supplementary Fig. 5. *In vitro* characterization of EMP induced by sh*CDH1* and ectopic *ZEB1* expression.

a-c. Representative immunofluorescence micrographs for CK8 (cyan) and E-cad (magenta), counterstained with DAPI (grey), in budding structures (**a**), at the leading edge (**b**) and next to blood vessel (**c**). Scale bars, 50 µm; inlet, 10 µm. **d.** Phase contrast micrograph of shSCR and sh*CDH1* MCF-7 cells; scale bars, 100 µm. **e.** Representative Western blot of total cell lysates from MCF-7 cells *in vitro* transduced with either shSCR or sh*CDH1*-encoding lentiviruses and probed for E-cad and Lamin B1; 4 biological replicates. **f.** Relative proliferation of shSCR and sh*CDH1* MCF-7 cells day 3 after seeding assessed by MTT assay. Data represent mean ± SD of 4 biological replicates. Two-tailed unpaired t test. **g.** Relative mRNA levels of marker genes in shSCR and sh*CDH1* MCF-7 cells. Data represent mean ± SEM of 3 biological replicates. Two-tailed unpaired t-test. **h.** Representative phase contrast micrograph of control and *ZEB1* MCF-7 cells; scale bars, 100 µm **i.** Western blot of ZEB1, E-CAD, ER, and Lamin B1 in control and *ZEB1* MCF-7 cells. **j.** Fold-change MTT absorbance of control and *ZEB1* MCF-7 cells over time. Data represent mean of 3 biological replicates ± SD. Two-way ANOVA, multiple comparison. **k.** Relative mRNA levels of marker genes in control and *ZEB1* MCF-7 cells, 3-4 biological replicates. Data represent mean ± SD of at least 3 biological replicates. One-way ANOVA. *, **, ***, ****, and n.s., represent $P<0.05$, 0.01, 0.001, 0.0001, and not significant, respectively.

Supplementary Figure 6

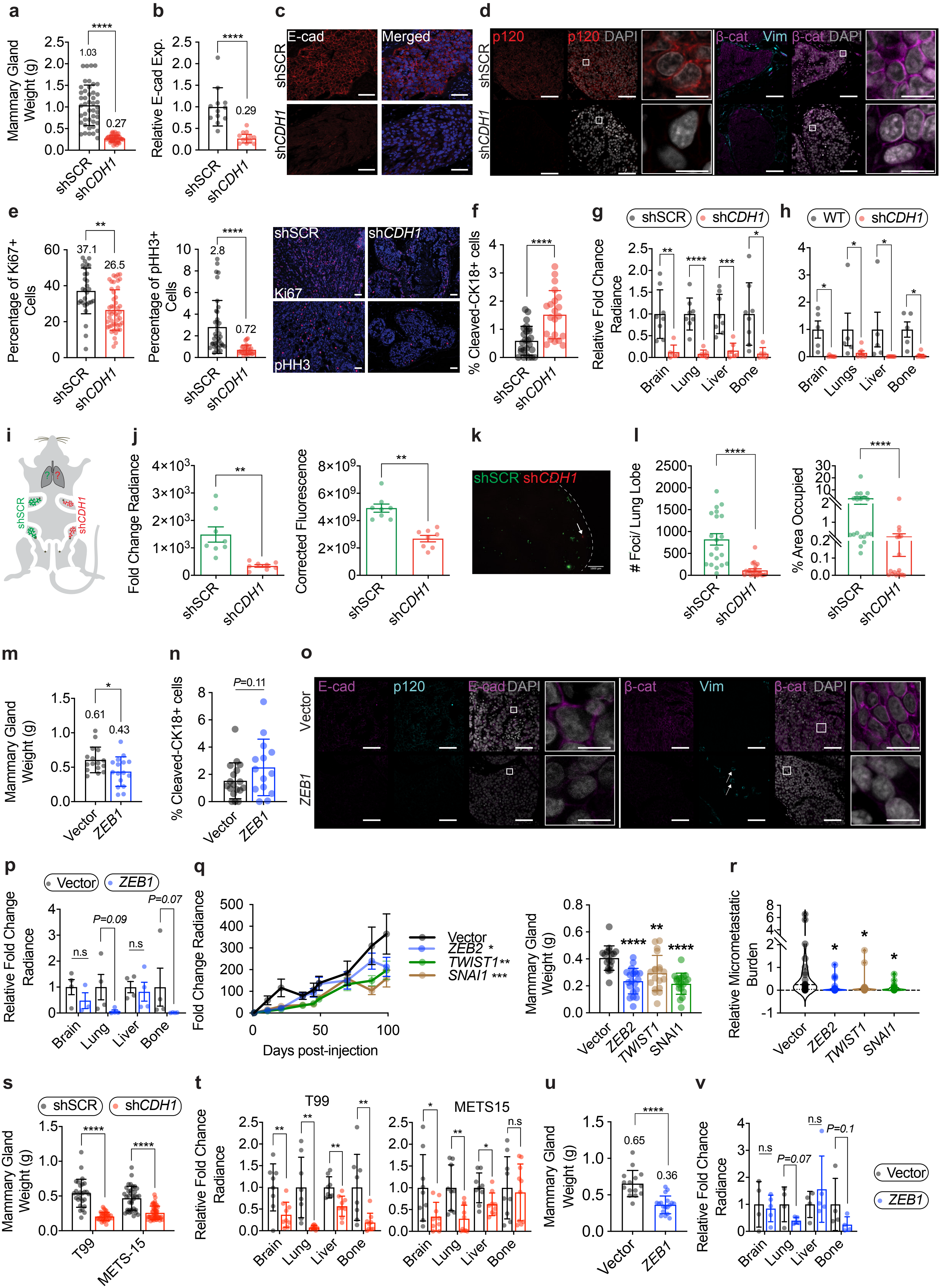

Supplementary Fig. 6. *In vivo* EMP characterization.

a. Weight of mammary glands xenografted with either shSCR or shCDH1 MCF-7 cells at end-point from 46 shSCR and 43 sh*CDH1* xenograft glands, total of 25 mice. Data represent mean ± SD, two-tailed unpaired Student's t-test. **b.** Relative E-cad levels by Western blot in 12 shSCR and sh*CDH1* xenograft mammary glands from at least 5 mice. Two-tailed unpaired Student's t-test. **c.** Representative IF staining of E-cad in MCF-7 shSCR and sh*CDH1* xenograft mammary glands from contralateral mice (n=4). Nuclei are counter-stained with DAPI. Scale bar, 100 µm. **d.** Representative IF micrographs of p120, β-catenin (β-cat), and Vim in MCF-7:shSCR and sh*CDH1* intraductal xenografts. Nuclei were counterstained with DAPI; scale bar, 50 µm; inlets, 10 µm. **e.** Percentage of Ki67 and pHH3-positive cells in contralaterally-engrafted shSCR and sh*CDH1* and their representative IFs. Scale bar, 100 µm. Each dot represents at least 100 cells per analyzed image from n=4 mice. Two-tailed paired Student's t-test. **f.** Percentage of cleaved-cytokeratin 18^+ cells (cCK-18+) in MCF-7:shSCR and sh*CDH1* xenografts from n=4 mice. Each dot represents at least 100 cells per analyzed image. Data represent mean ± SD. Two-tailed Student's t-test. **g.** Relative fold-change in ex vivo organ bioluminescence in 7 shSCR and 8 sh*CDH1* tumor-bearing mice. Data represent mean ± SD, two-tailed multiple unpaired t-tests. **h.** Relative fold-change in ex vivo organ bioluminescence from 5 WT and 5 sh*CDH1* MCF-7 tumor-bearing mice with similar primary tumor bioluminescence. Data represent mean ± SD, two-tailed paired t-test. **i.** Scheme showing the contralateral engraftment of GFP^+ shSCR (left) and RFP^+ GFP^+sh*CDH1* (right) MCF-7 cells. **j.** Bar plots showing the bioluminescence fold-change (left) and corrected fluorescence (right) as detected by fluorescence stereomicroscopy at end-point of 8 contralateral shSCR:GFP and sh*CDH1*:RFP xenografts. Data represent mean ± SD, two-tailed paired t-test. **k.** Representative fluorescence stereograph of green shSCR and red sh*CDH1* cells contralaterally-engrafted. **l.** Quantification of total red and green MCF-7 foci (left) and their percentage area (right) occupied in shSCR and sh*CDH1*-bearing mice. Data represent mean ± SD of 20 lobes from 4 mice, two-tailed paired t-test. **m.** Weight of xenograft glands at end-point from 16 pairs of control and *ZEB1*-MCF-7 xenograft glands. Data represent mean ± SD, unpaired Student's t-test. **n.** Percentage of cleaved CK18 in MCF-7:Control and *ZEB1* xenografts from 3 mice. Each dot represents at least 100 cells per analyzed image. Data represent mean ± SD. Two-tailed unpaired Student's t-test. **o.** Representative IF micrographs of p120, β-cat, and Vim in MCF-7:Control and *ZEB1* intraductal xenografts. Nuclei were counterstained with DAPI; scale bar, 50 µm; inlets, 10 µm. Arrows point to Vim^+ MCF-7:*ZEB1* cells. **p.** Relative fold change in *ex vivo* organ bioluminescence from 4 mice, each. Data represent mean ± SD, two-tailed multiple unpaired t-tests. **q.** Growth curve and weight of 17, 20, 19, and 19 mammary glands xenografted with Control, *ZEB2*-, *TWIST1*-, and *SNAI1*-overexpressing MCF-7 cells, respectively, in 5 mice per condition. Data represent mean ± SEM, Two-way and One-way ANOVA tests, respectively. **r.** Violin plots showing the relative micro-metastatic burden in mice bearing Control, *ZEB2*-, *TWIST1*-, and *SNAI1*-overexpressing MCF-7 cells; 5 mice per condition. **s.** Weight of 28 shSCR and 34 sh*CDH1* T99 xenograft glands (8 and 9 mice, respectively) and 43 shSCR and 47 sh*CDH1* METS15 xenograft glands (9 mice for each) at end-point. Data represent mean ± SD, two-tailed unpaired Student's t-test. **t.** Relative fold change in *ex vivo* organ bioluminescence in 8 shSCR and 9 sh*CDH1* T99 xenografts-bearing mice and in 9 shSCR and 9 sh*CDH1* METS15 xenografts-bearing mice, Data represent mean ± SD, two-tailed multiple t-tests. **u.** Mammary gland weight at end-point of T99 control and *ZEB1* intraductal xenografts. Data represent mean ± SD of 15 and 18 xenograft glands, respectively. **v.** Relative fold change in *ex vivo* organ bioluminescence in 4 control and 5 *ZEB1*-overexpressing T99 xenografts-bearing mice, respectively. Multiple unpaired t-tests. *, **, ***, ***, and n.s represent *P*<0.05, 0.01, 0.001, 0.0001, and not significant, respectively.

Supplementary Figure 7

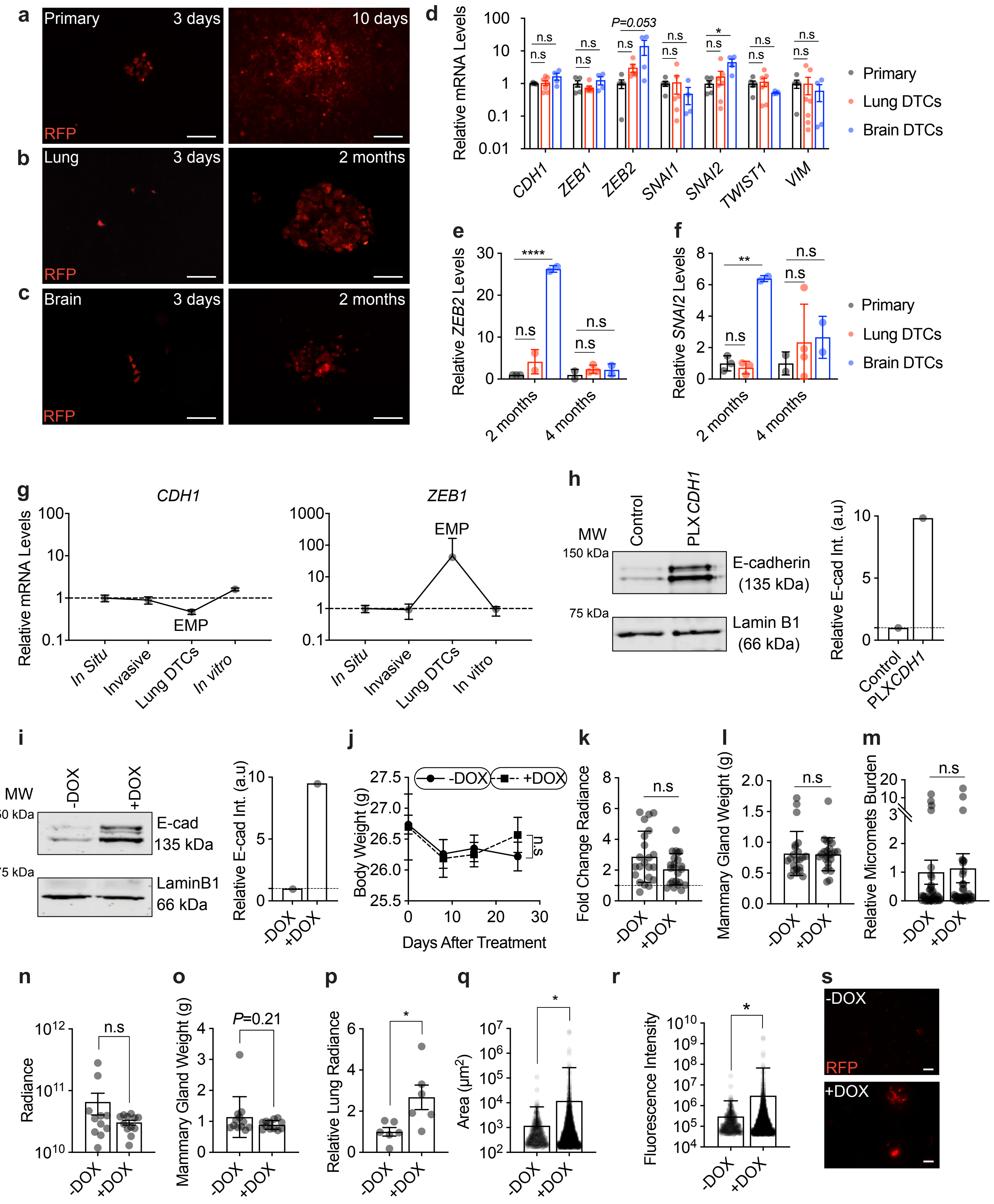

Supplementary Fig. 7. EMP *in vitro*.

a. *Ex vivo* culture of RFP$^+$ MCF-7 primary tumor cells 3 and 10 days after plating. Scale bar, 200 μm. **b, c**. *Ex vivo* culture of RFP$^+$ MCF-7 lung **(b)** and brain **(c)** DTCs 3 days and 2 months after plating. Scale bar, 200 μm. **d.** Relative mRNA levels of selected genes in *in vitro* cultured MCF-7 primary tumor cells and lung and brain DTCs up to 4 months in culture, normalized to the geometric mean of *GAPDH* and *HPRT*. Data represent mean ± SD from n=5 control, 7 lung, and 4 brain replicates. **e.** Relative *ZEB2* and **f.** *SNAI2* levels in primary, lung and brain DTCs cultured for 2 and 4 months. Data represent mean ± SD from at least 2 biological replicates, two-way ANOVA. **g.** Summary of the relative expression levels of *CDH1* (left) and *ZEB1* (right) in intraductally grafted MCF-7 cells either in *in situ*, invasive tumors, DTCs or after 2 months of *in vitro* culture. Data represent mean ± SD; for *in situ* and invasive 6 glands from 3 mice, for DTCs 8 mice, and for *in vitro* 3 biological replicates. EMP: Epithelial-Mesenchymal Plasticity; MET: EMP-Epithelial Transition. **h, I**. Western blot validation and quantification of E-cad overexpression in MCF-7 cells **(h)** and E-cad induction by 2 μg/ml in MCF-7:*E-cad^{IND}* cells *in vitro* **(i)**. **j.** Body weight of mice treated with or without DOX across 4 weeks. Data represent mean ± SEM from 5 mice in each cohort. Two-way ANOVA. **k-m**. Bar plot showing the fold change radiance relative to day 0 of DOX administration **(k)**, mammary gland weight **(l)**, and micro-metastatic burden **(m)** of MCF-7 intraductal xenografts treated with (23 mammary glands from 9 mice) or without (22 mammary glands from 9 mice) 45 days after treatment. Data represent mean ± SD. Two-tailed unpaired t-test (k,l) and two-tailed Mann-Whitney test (m). **n, o.** Bar plots showing the log10 radiance values **(n)** and mammary gland weight **(o)** at end-point of T47D:RFP-Luc:E-cadIND intraductal xenografts treated with or without DOX. Data represent mean ± SEM of 12 mammary glands from 6 mice. Two-tailed Student t-test. **p.** Bar plot showing the relative *ex vivo* lung radiance in T47D:RFP-Luc:E-cadIND intraductal xenografts treated with or without DOX. Data represent mean ± SEM from 6 mice. **q, r.** Bar plots showing the area **(q)** and fluorescence intensity **(r)** of lung lesions in T47D:RFP-Luc:E-cadIND-bearing mice (n=6) treated with or without DOX. Each dot represents areas or fluorescence intensity of metastatic cells from analyzed image. Data represent mean ± SD. **s.** Fluorescence stereo micrographs of lung lesions in -DOX (top panel) and +DOX (bottom panel) treated mice. Scale bar, 500 μm.

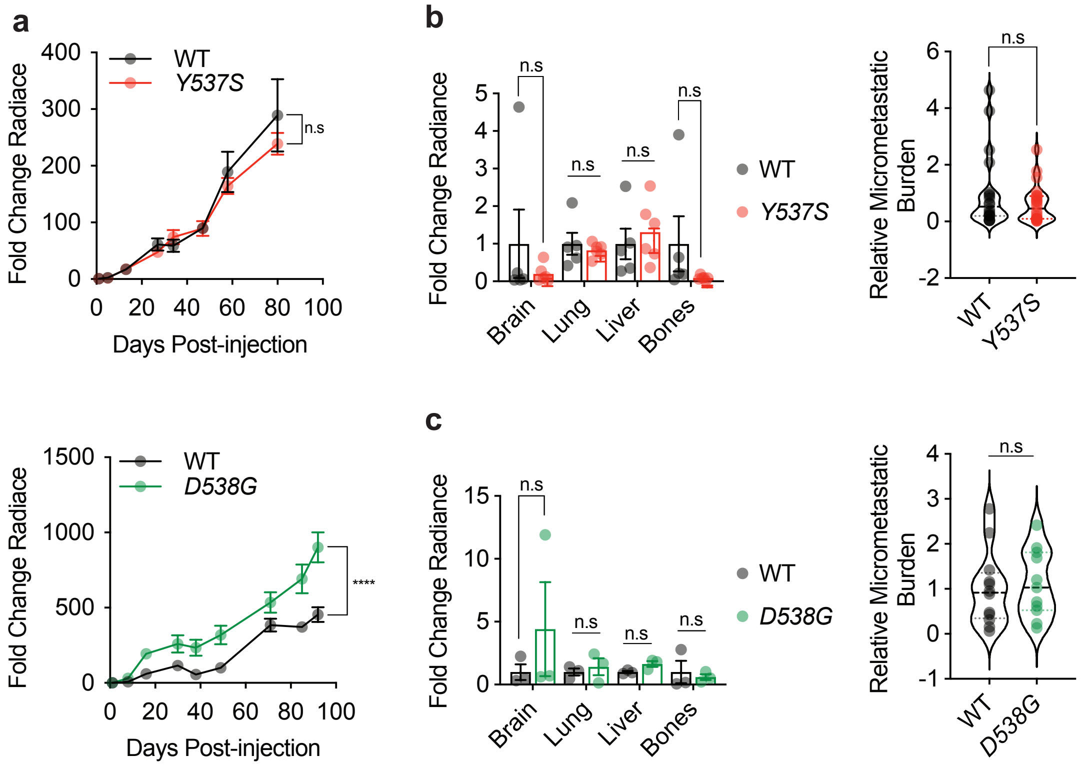

Supplementary Fig. 8. Growth and metastasis of *ESR1*^{mutant} MCF-7 cells *in vivo*

a. Graph showing the fold-change radiance over time for intraductal MCF-7:*WT* and *ESR1*^{Y537S} (top panel), and *WT* and *ESR1*^{D538G} (bottom panel) xenografts. Data represent mean ± SEM from n=16, 12, 12, 12 xenografts, respectively. Two-way ANOVA. **b.** *Ex vivo* radiance (left panel) and relative micro-metastatic burden (right panel) in *WT* and *ESR1*^{Y537S} MCF-7 xenografted mice. Data represent mean ± SEM. Multiple unpaired t-test. **c.** *Ex vivo* radiance (left panel) and relative micro-metastatic burden (right panel) in *WT* and *ESR1*^{D538G} from n=3 mice each. Data represent mean ± SEM. Two-tailed Welch's test.

Supplementary Figure 9

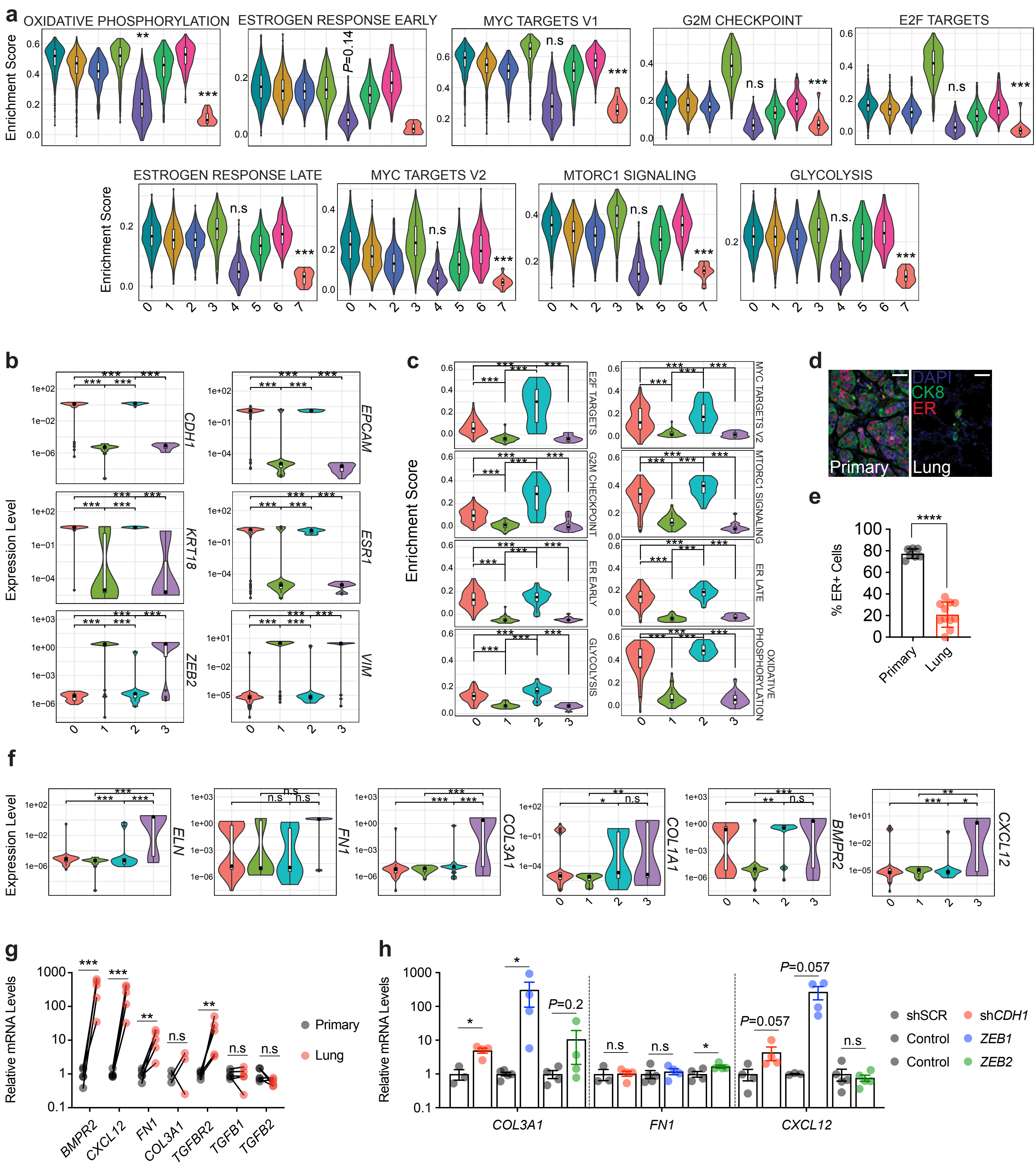

Supplementary Fig. 9. Heterogeneity of ER⁺ primary tumor cells and lung DTCs.

a. Violin plots showing differences of enrichment score in the illustrated gene sets of the Hallmark database across cell clusters in the primary tumor. Boxplots inside each violin describe the interquartile range, bold dots indicate median values. Statistical significance was assessed by fitting a generalized linear mixed model and computing the estimated marginal means across all the identified cell clusters. Total n tumor cells in the primary tumor = 2558: n cluster 0 = 602, n cluster 1 = 458, n cluster 2 = 409, n cluster 3 = 355, n cluster 4 = 327, n cluster 5 = 226, n cluster 6 = 166, n cluster 7 = 15. Boxplot whiskers show minimum and maximum values. Tukey's method was used to assess adjusted p-values. The statistical significance illustrated on the plots refers to all possible pairwise comparisons with respect to cluster 7, unless otherwise indicated. **b.** Violin plots showing differences in expression levels of the epithelial (*CDH1*, *EPCAM*, *KRT18*, *ESR1*) and mesenchymal (*ZEB2*, *VIM*) markers in the identified clusters of lung DTCs. Total n lung DTCs = 194: n cluster 0 = 109, n cluster 1 = 39, n cluster 2 = 27, n cluster 3 = 19. Boxplots inside each violin describe the interquartile range, bold dots indicate median values. Boxplot whiskers show minimum and maximum values. Statistical significance was assessed by two-sided Wilcoxon rank sum test, adjusted *p*-values were estimated with the Holm's correction method. **c.** Violin plots showing differences of enrichment score in the illustrated gene sets of the Hallmark database across cell clusters in the lung DTCs. For number of cells analyzed, refer to (b)**.** Boxplots inside each violin describe the interquartile range, bold dots indicate median. Boxplot whiskers show minimum and maximum values. Statistical significance was assessed by fitting a generalized linear mixed model and computing the estimated marginal means across all the identified cell clusters. Tukey's method was used to assess adjusted *p*-values. **d.** Representative IF micrographs showing anti-CK8 (green) and anti-ER (red) antibodies on histological sections from primary tumor (left) and lung DTCs (right). Scale bar, 50 µm. **e.** Bar plot showing the percentage of ER⁺ cells in MCF-7 intraductal xenografts and lung DTCs from at least 3 mice. Scale bar, 20 µm. Data represent mean ± SD; 130,294 primary tumor cells and 2,207 lung DTCs were analyzed. Two-tailed Student's unpaired t-test. **f.** Violin plots showing differences in expression levels of selected tumor-derived ECM and stromal crosstalk markers in the identified clusters in lung DTCs. For number of cells analyzed, refer to (b). Boxplots inside each violin describe the interquartile range, bold dots indicate median values. Boxplot whiskers show minimum and maximum values. Statistical significance was assessed by two-sided Wilcoxon rank sum test, adjusted *p*-values were estimated with the Holm's correction method. **g.** Relative mRNA levels in matched METS15 primary tumor cells and lung DTCs (n=5 mice) normalized to the geometric mean of *GAPDH* and *HPRT*. Statistical significance was assessed by two-tailed paired t-test. **h.** Relative mRNA levels of dormancy-related genes in controls, *ZEB1*-, and *ZEB2*-expressing MCF-7 cells in vitro. Data represent mean ± SEM from at least 3 biological replicates. Statistical significance was assessed by two-tailed student's t-test. *, **, ***, ***, and n.s. repre-

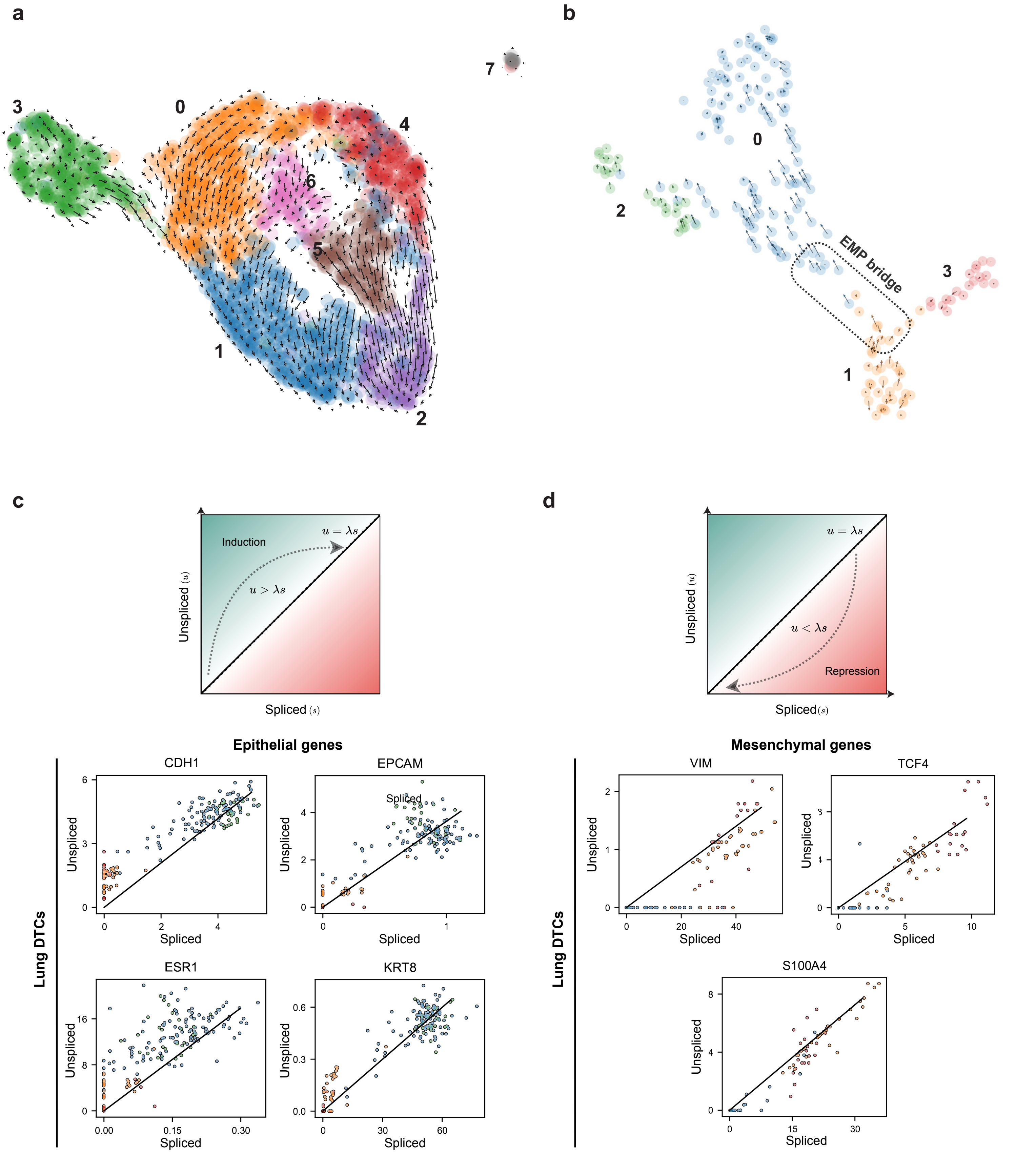

Supplementary Figure 10. RNA velocity points to MET in lung DTCs.
a, b. UMAP showing the steady-state RNA velocity arrows in MCF-7 cells at the primary site **(a)** and in the lungs **(b)** of intraductally xenografted mice. Velocity estimates projected onto the two-dimensional UMAP of data shown in Fig. 7a,b. Velocity vectors indicate positive velocity, directional flow, from one cell state to another and show directionality from Cluster 1 to Cluster 0. The EMP bridge refers to cells transitioning from an inactive to an active state, and is represented by a dashed rectangle. **c, d.** Unspliced–spliced phase portraits of lung DTCs. In the schemes the linear line of slope represents the steady-state and the clockwise arc depicts the changes in unspliced versus spliced RNA in a cell at a given time. Phase plots of epithelial genes *CDH1*, *EPCAM*, *ESR1*, and *KRT18* show induction **(c)** and the mesenchymal genes *VIM*, *TCF-4*, and *S100A4* show repression **(d)**.

Supplementary Figure 11

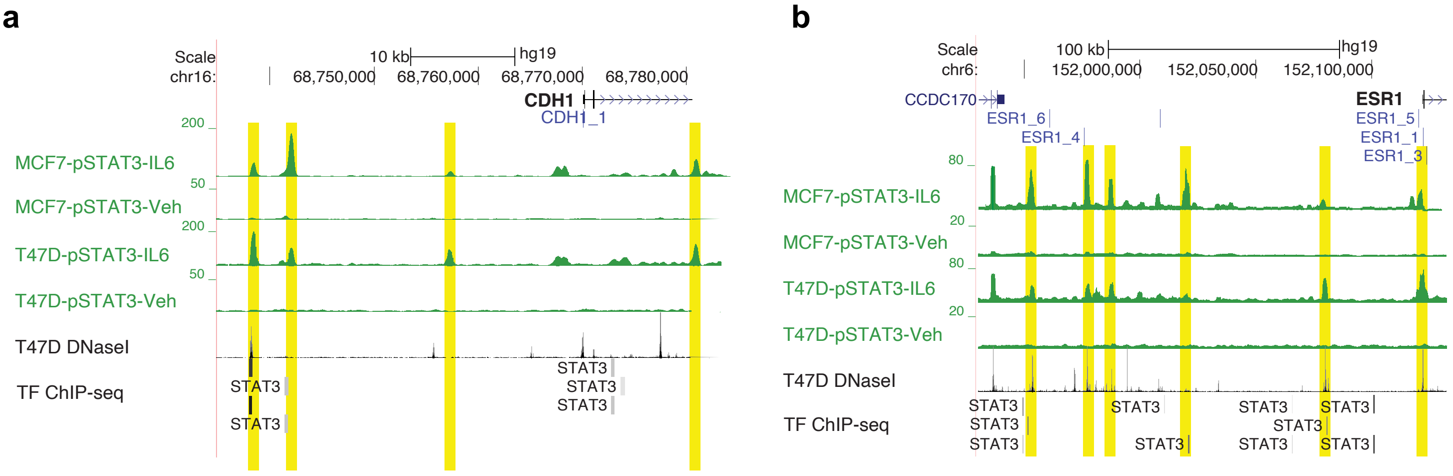

Supplementary Figure 11. pSTAT3 binding to *CDH1* and *ESR1* promoter and enhancer regions.
a. UCSC genome browser screenshot showing IL6-induced pSTAT3 binding at *CDH1* promoter and enhancer regions. The browser window shows regions, highlighted in yellow, that are bound by pSTAT3 upon IL6 stimulation in MCF-7 and T47D cells (Siersbæk et al., 2020). The other tracks shown are from the UCSC genome browser database: UCSC genes (multiple sources), EPD new promoters (Meylan et al., 2020), T47D DNaseI from ENCODE (Sabo et al., 2004), ChIP-seq clusters from ENCODE/Factorbook (Wang et al., 2013). **b.** UCSC genome browser screenshot showing IL6-induced pSTAT3 binding at *ESR1* promoter and enhancer regions. The browser window show regions, highlighted in yellow, that are bound by phosphorylated pSTAT3 upon IL6 stimulation in two breast cancer cell lines, MCF-7, and T47D. The other tracks shown are from the UCSC genome browser database: UCSC genes (multiple sources), EPDnew promoters, T47D DNaseI from ENCODE, and ChIP-seq clusters from ENCODE/Factorbook.

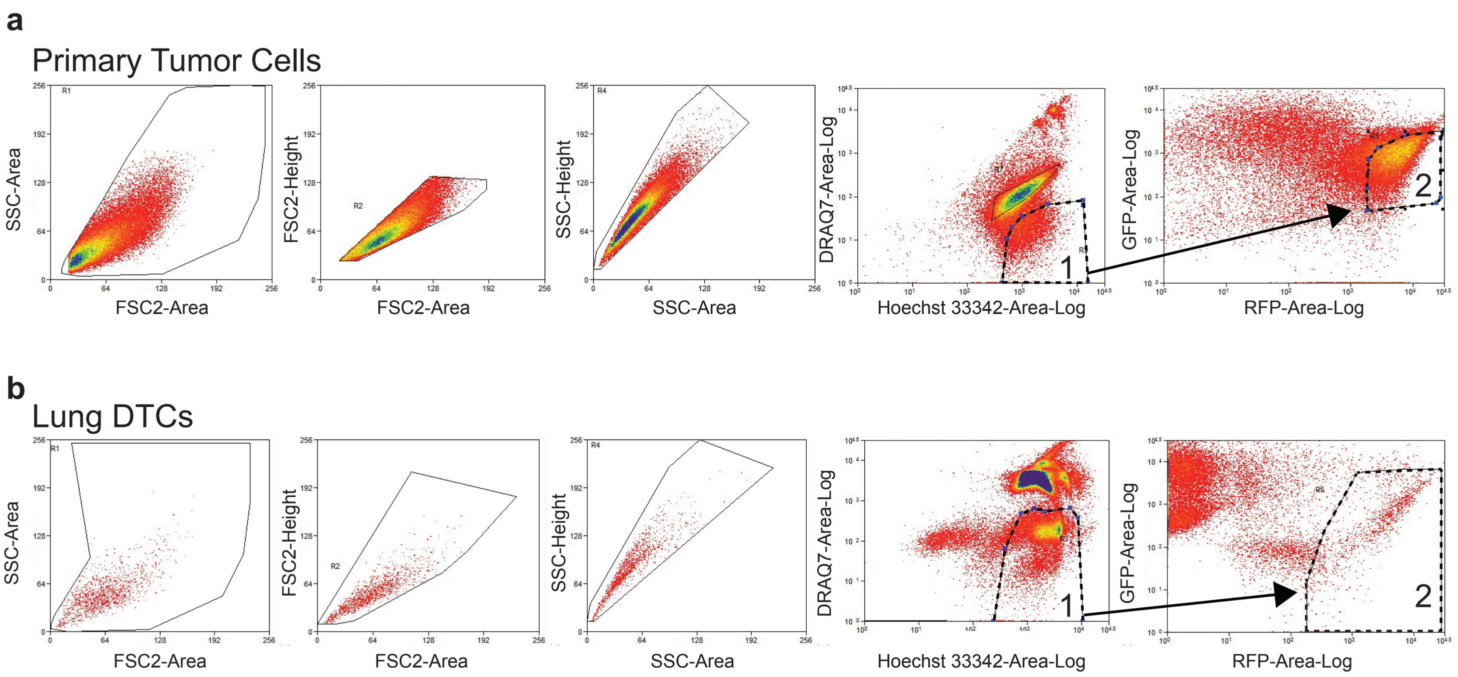

Supplementary Figure 12. Gating strategy for primary tumor cell and lung DTC analysis.
a, b. GFP-RFP+DRAQ7-Hoechst 33342+ primary tumor cells **(a)** and lung DTCs **(b)** were gated and sorted.

Supplementary Table 1. Total number of cells scored for Ki67 in matched primary and lung metastases in ER+ and TNBC intraductal xenografts.

	Primary	Lung
MCF-7	38,847	1,909
T47D	43,162	548
METS15	62647	1087
BT20	30,459	2,593

Supplementary Table 2. List of antibodies used in this study. IF: immunofluorescence, WB: Western blot.

Antibody	Source	Identifier	Clone	Usage	Dilution
CK8	BioLegend	MMS-162P-250	1E8	IF	1 in 400
DsRed	MBL Int.	PM005	N/A	IF	1 in 500
E-cadherin	Cell Signaling	3195S	24E10	WB, IF	1 in 100
ER	Ventana Medical Systems	790-4324	SP-1	WB	1 in 200
GFP	Santa Cruz	sc-9996	B-2	IF	1 in 500
Ki67	ORIGENE	TA801577	N/A	IF	1 in 100
	ThermoFisher Scientific	MA5-14520	SP-6	IF	1 in100
Lamin B1	Abcam	AB16048	N/A	WB	1 in 1000
pHH3 (ser10)	Millipore	06-570	3H10	IF	1 in 500
p27 Kip1	Cell Signaling	3686	D69C12	IF	1 in 100
Vimentin D21H3 XP	Cell Signaling	5741S	N/A	IF	1 in 500
p120	BD Biosciences	610133	N/A	IF	1 in 100
β-catenin	BD Biosciences	610154	N/A	IF	1 in 100
Cleaved Cytokeratin-18	Roche	12140322001	M30	IF	1 in 200
Zeb1	Novus Biologicals	NBP1-05987	N/A	WB	1 in 500
Mouse Alexa 488	ThermoFisher Scientific	A-11029	N/A	IF	1 in 500
Mouse Alexa 568	ThermoFisher Scientific	A-10037	N/A	IF	1 in 500
Mouse Alexa 647	ThermoFisher Scientific	A-31571	N/A	IF	1 in 500
Rabbit Alexa 488	ThermoFisher Scientific	A-21206	N/A	IF	1 in 500
Rabbit Alexa 568	ThermoFisher Scientific	A-10042	N/A	IF	1 in 500
Rabbit Alexa 647	ThermoFisher Scientific	A-31573	N/A	IF	1 in 500
Rat Alexa 647	ThermoFisher Scientific	A-21247	N/A	IF	1 in 500

Supplementary Table 3. List of primers used in this study.

Gene	Forward Primer	Reverse Primer	Reference
CDH1	5'-TGC CCA GAA AAT GAA AAA GG-3'	5'-GTG TAT GTG GCA ATG CGT TC-3'	1
CDH2	5'-ACA GTG GCC ACC TAC AAA GG-3'	5'-CCG AGA TGG GGT TGA TAA TG-3'	1
CDH3	5'-ATC ATC GTG ACC GAC CAG AAT-3'	5'-GAC TCC CTC TAA GAC ACT CCC-3'	N/A
ZEB1	5'-ACC TGC CAA CAG ACC AGA CAG TGT-3'	5'-CCT GAC CTT CAG GCC CCA GGA-3'	N/A
ZEB2	5'-TTT CAG GGA GAA TTG CTT GA-3'	5'-CAC ATG CAT ACA TGC CAC TC-3'	2
	5'-AAT GCA CAG AGT GTG GCA AGG C-3'	5'-CTG CTG ATG TGC GAA CTG TAG G-3'	N/A
SNAI1	5'-GGA GTC CGC AGT CTT ACG AG-3'	5'-TCT GAA GAA CCT GGT AGA GG-3'	1
SNAI2	5'-CTG GGC GCC CTG AAC ATG CAT-3'	5'-GGC TTC TCC CCC GTG TGA GTT CTA-3'	3
VIM	5'- GCA AAG ATT CCA CTT TGC GT-3'	5'-GAA ATT GCA GGA GGA GAT GC-3'	4
	5'-GAG AAC TTT GCC GTT GAA GC-3'	5'-GCT TCC TGT AGG TGG CAA TC-3'	N/A
TWIST1	5'-CAT CCT CAC ACC TCT GCA TT-3'	5'-GGC CAG TTT GAT CCC AGT AT-3'	1
GAPDH	5'-AGG GCT GCT TTT AAC TCT GCT-3'	5'-CCC CAC TTG ATT TTG GAG GGA-3'	N/A
HPRT	5'-TAG AAG GCC TTG TGC TCA CC-3'	5'-TCT GCT CTG ACT TTA GCA CCT G-3'	N/A
MKI67	5'-CGG ACT TTG GGT GCG ACT T-3'	5'-GTC GAC CCC GCT CCT TTT-3'	N/A
TBP	5'-TAG AAG GCC TTG TGC TCA CC-3'	5'-TCT GCT CTG ACT TTA GCA CCT G-3'	N/A
ESR1	5'-GGA GAT CTT CGA CAT GCT GC-3'	5'-GCC ATC AGG TGG ATC AAA GT-3'	N/A
PgR	5'-AAA CTG CCC AGC ATG TCG TCT-3'	5'-GCT CTG GTT AGG AAG GCC CA-3'	N/A
AR	5'-GAG AAC TTT GCC GTT GAA GC-3'	5'-GCT TCC TGT AGG TGG CAA TC-3'	N/A
Epcam	5'-ACG CGT TGT GAT CTC CTT CT-3'	5'-GCT CTG AGC GAG TGA GAA CC-3'	N/A
BMPR2	5'-CCA AGA GTG TCA CTA TGA AG-3'	5'-TGA ATG AGG TGG ACT GAG TG-3'	5
CXCL12	5'-CTT TAG CTT CGG GTC AAT GC-3'	5'-TCA GCC TGA GCT ACA GAT GC-3'	6
FN1	5'-CGG TGG CTG TCA GTC AAA G-3'	5'-AAA CCT CGG CTT CCT CCA TAA-3'	N/A
COL3A1	5'-GGA GCT GGC TAC TTC TCG C-3'	5'-GGG AAC ATC CTC CTT CAA CAG-3'	7
TGFβR2	5'-ATG ACA TCT CGC TGT AAT GC-3'	5'-GGA TGC CCT GGT GGT TGA-3'	8
TGFβ-1	5'-TCG CCA GAG TGG TTA TCT T-3'	5'-TAG TGA ACC CGT TGA TGT CC-3'	8
TGFβ-2	5'-ACA CTC AGC ACA GCA GGG TCC T-3'	5'-TTG GGA CAC GCA GCA AGG AGA AG-3'	8

Supplementary Table 4. Table describing the GSEA for hallmarks in active and inactive lung clusters. P value was determined using Wilcoxon test.

	T_statistic	*P* value	mean_Active	mean_Inactive	FDR
HALLMARK_XENOBIOTIC_METABOLISM	27.13627239	1.34E-67	0.145625866	-0.063900344	7.51E-66
HALLMARK_DNA_REPAIR	27.01740863	3.87E-67	0.203007246	-0.072372564	2.13E-65
HALLMARK_ESTROGEN_RESPONSE_LATE	26.92421244	3.15E-65	0.139083562	-0.058611904	1.7E-63
HALLMARK_OXIDATIVE_PHOSPHORYLATION	25.87574078	1.07E-63	0.411270078	0.058757526	5.65E-62
HALLMARK_CHOLESTEROL_HOMEOSTASIS	25.30720878	4.55E-63	0.246081171	-0.001121665	2.37E-61
HALLMARK_ESTROGEN_RESPONSE_EARLY	25.29073596	2.23E-61	0.135427423	-0.049195161	1.14E-59
HALLMARK_ADIPOGENESIS	23.46425557	6.57E-58	0.221204798	0.059650804	3.28E-56
HALLMARK_MTORC1_SIGNALING	22.13094882	2.35E-54	0.32815738	0.119169535	1.13E-52
HALLMARK_MYC_TARGETS_V1	22.50541982	1.31E-52	0.528352323	0.203671011	6.17E-51
HALLMARK_ANDROGEN_RESPONSE	20.85140591	1.06E-47	0.298195247	0.129878642	4.9E-46
HALLMARK_UNFOLDED_PROTEIN_RESPONSE	20.27703693	1.17E-47	0.293346258	0.132537109	5.28E-46
HALLMARK_UV_RESPONSE_UP	19.5041809	2.53E-47	0.190559909	0.068973698	1.11E-45
HALLMARK_KRAS_SIGNALING_DN	-19.26524091	1.66E-46	-0.029520723	0.107809075	7.13E-45
HALLMARK_FATTY_ACID_METABOLISM	18.54462184	1.25E-44	0.165883516	0.03978037	5.23E-43
HALLMARK_REACTIVE_OXYGEN_SPECIES_PATHWAY	20.03969808	1.03E-43	0.2743747	0.00219448	4.12E-42
HALLMARK_P53_PATHWAY	17.57562	7.94E-42	0.185047238	0.093091717	3.1E-40
HALLMARK_GLYCOLYSIS	16.97352493	5.43E-40	0.140620661	0.060477892	2.06E-38
HALLMARK_APICAL_JUNCTION	16.21995525	7.92E-38	0.07591333	-0.056442505	2.93E-36
HALLMARK_APICAL_SURFACE	16.64817365	5.78E-37	0.075142291	-0.138287087	2.08E-35
HALLMARK_APOPTOSIS	16.41228061	2.56E-36	0.251002242	0.113334534	8.71E-35
HALLMARK_PROTEIN_SECRETION	15.56678136	1.73E-34	0.244799348	0.059182719	5.7E-33
HALLMARK_HYPOXIA	13.64397979	1.36E-29	0.209611991	0.104448007	4.37E-28
HALLMARK_MYC_TARGETS_V2	13.10354464	2.55E-27	0.150970278	0.015803402	7.89E-26
HALLMARK_HEME_METABOLISM	12.62300709	8.44E-27	0.101505042	0.036431864	2.53E-25
HALLMARK_E2F_TARGETS	12.75827996	2.09E-26	0.108343649	-0.036630815	6.05E-25
HALLMARK_G2M_CHECKPOINT	12.00065625	4.84E-25	0.129495508	0.009707721	1.36E-23
HALLMARK_PANCREAS_BETA_CELLS	12.44696909	4.43E-22	0.26632799	0.053209898	1.2E-20
HALLMARK_PI3K_AKT_MTOR_SIGNALING	11.33791884	4.89E-22	0.204080697	0.095991064	1.27E-20
HALLMARK_UV_RESPONSE_DN	-9.896942105	6.63E-18	0.143881219	0.214591024	1.66E-16
HALLMARK_IL6_JAK_STAT3_SIGNALING	8.934978003	9.47E-16	0.053748952	-0.037061019	2.27E-14
HALLMARK_TNFA_SIGNALING_VIA_NFKB	8.813854175	1.04E-15	0.287715339	0.171208107	2.4E-14
HALLMARK_TGF_BETA_SIGNALING	8.820899403	5.51E-15	0.269886742	0.156333363	1.21E-13
HALLMARK_MITOTIC_SPINDLE	8.028589444	1.1E-13	0.031235865	-0.026573045	2.31E-12
HALLMARK_MYOGENESIS	8.101949154	1.96E-13	0.03503082	-0.03029448	3.93E-12
HALLMARK_PEROXISOME	7.508383634	2.78E-12	0.146268121	0.101610686	5.27E-11
HALLMARK_INTERFERON_GAMMA_RESPONSE	5.675014038	5.18E-08	0.122128822	0.080669294	0.000000881
HALLMARK_KRAS_SIGNALING_UP	-5.472116668	0.000000138	0.07623028	0.114904732	0.0000022
HALLMARK_COAGULATION	4.363195133	0.0000254	0.113195062	0.070069627	0.000381392

HALLMARK_IL2_STAT5_SIGNALING	3.783398994	0.000210363	0.130113726	0.106886431	0.00294508
HALLMARK_ANGIOGENESIS	-3.774917911	0.000251464	0.135751663	0.192820787	0.003269038
HALLMARK_EPITHELIAL_MESENCHYMAL_TRANSITION	-3.662090963	0.00040027	0.147651348	0.187840185	0.004803234
HALLMARK_ALLOGRAFT_REJECTION	3.410536448	0.000948876	0.20536872	0.172430041	0.010437637
HALLMARK_HEDGEHOG_SIGNALING	3.045830629	0.002877473	-0.072175541	-0.116551231	0.028774727
HALLMARK_COMPLEMENT	2.124884638	0.035998895	0.154132642	0.13609099	0.323990053
HALLMARK_SPERMATOGENESIS	-1.887919672	0.060560729	0.051929655	0.063481819	0.484485832
HALLMARK_NOTCH_SIGNALING	-1.543790295	0.125315194	0.142671738	0.159894906	0.751891165
HALLMARK_WNT_BETA_CATENIN_SIGNALING	-0.954936273	0.341141968	0.033548119	0.043621841	1
HALLMARK_INFLAMMATORY_RESPONSE	-0.767926305	0.443691694	0.076529051	0.083240212	1
HALLMARK_BILE_ACID_METABOLISM	0.566943693	0.571443585	0.059022086	0.055469546	1
HALLMARK_INTERFERON_ALPHA_RESPONSE	-0.091976225	0.926813161	0.099186039	0.099888083	1

References:

1. Mani, S. A., Guo, W., Liao, M. J., Eaton, E. N., Ayyanan, A., Zhou, A. Y., ... & Weinberg, R. A. (2008). The epithelial-mesenchymal transition generates cells with properties of stem cells. Cell , 133 (4), 704-715.

2. Nam, E. H., Lee, Y., Park, Y. K., Lee, J. W., & Kim, S. (2012). ZEB2 upregulates integrin α5 expression through cooperation with Sp1 to induce invasion during epithelial–mesenchymal transition of human cancer cells. Carcinogenesis, 33(3), 563-571.

3. Zheng, H., Shen, M., Zha, Y. L., Li, W., Wei, Y., Blanco, M. A., Ren, G., Zhou, T., Storz, P., Wang, H. Y., and Kang, Y. (2014). PKD1 phosphorylation-dependent degradation of SNAIL by SCFFBXO11 regulates epithelial-mesenchymal transition and metastasis. Cancer Cell. 26, 358-373.

4. Chen, P. Y., Qin, L., Barnes, C., Charisse, K., Yi, T., Zhang, X., ... & Simons, M. (2012). FGF regulates TGF-β signaling and endothelial-to-mesenchymal transition via control of let-7 miRNA expression. Cell reports, 2(6), 1684-1696.

5. Kobayashi, A., Okuda, H., Xing, F., Pandey, P. R., Watabe, M., Hirota, S., ... & Watabe, K. (2011). Bone morphogenetic protein 7 in dormancy and metastasis of prostate cancer stem-like cells in bone. Journal of Experimental Medicine, 208(13), 2641-2655.

6. Li, T., Li, H., Wang, Y., Harvard, C., Tan, J. L., Au, A., ... & You, L. (2011). The expression of CXCR4, CXCL12 and CXCR7 in malignant pleural mesothelioma. The Journal of pathology, 223(4), 519-530.

7. Nagar, H., Kim, S., Lee, I., Kim, S., Choi, S. J., Piao, S., ... & Kim, C. S. (2021). Downregulation of CR6-interacting factor 1 suppresses keloid fibroblast growth via the TGF-β/Smad signaling pathway. Scientific Reports, 11(1), 1-13.

8. Chen, C., Zhao, K. N., Masci, P. P., Lakhani, S. R., Antonsson, A., Simpson, P. T., & Vitetta, L. (2015). TGFβ isoforms and receptors mRNA expression in breast tumours: prognostic value and clinical implications. BMC cancer, 15(1), 1-12.
