# Supplementary material for: Epithelial-mesenchymal plasticity determines estrogen receptor positive breast cancer dormancy and epithelial reconversion drives recurrence
Source: Nat Commun. 2022 Aug 25;13:4975. doi: 10.1038/s41467-022-32523-6 (PMC9411634; doi:10.1038/s41467-022-32523-6)
Supplement: Supplementary file 2 — Reporting Summary [file 41467_2022_32523_MOESM2_ESM.pdf]

nature research

Corresponding author(s): Cathrin Brisken, MD, PhD

Last updated by author(s): 22/07/17

Reporting Summary

Nature Research wishes to improve the reproducibility of the work that we publish. This form provides structure for consistency and transparency in reporting. For further information on Nature Research policies, see our Editorial Policies and the Editorial Policy Checklist.

Statistics

For all statistical analyses, confirm that the following items are present in the figure legend, table legend, main text, or Methods section.

n/a	Confirmed	
☐	☒	The exact sample size (n) for each experimental group/condition, given as a discrete number and unit of measurement
☐	☒	A statement on whether measurements were taken from distinct samples or whether the same sample was measured repeatedly
☐	☒	The statistical test(s) used AND whether they are one- or two-sided<br>*Only common tests should be described solely by name; describe more complex techniques in the Methods section.*
☒	☐	A description of all covariates tested
☐	☒	A description of any assumptions or corrections, such as tests of normality and adjustment for multiple comparisons
☐	☒	A full description of the statistical parameters including central tendency (e.g. means) or other basic estimates (e.g. regression coefficient) AND variation (e.g. standard deviation) or associated estimates of uncertainty (e.g. confidence intervals)
☐	☒	For null hypothesis testing, the test statistic (e.g. F, t, r) with confidence intervals, effect sizes, degrees of freedom and P value noted<br>*Give P values as exact values whenever suitable.*
☒	☐	For Bayesian analysis, information on the choice of priors and Markov chain Monte Carlo settings
☒	☐	For hierarchical and complex designs, identification of the appropriate level for tests and full reporting of outcomes
☒	☐	Estimates of effect sizes (e.g. Cohen's d, Pearson's r), indicating how they were calculated

Our web collection on statistics for biologists contains articles on many of the points above.

Software and code

Policy information about availability of computer code

Data collection

No software was used for data collection.

Data analysis

PRISM (version 8) https://www.graphpad.com/
Fiji/ImageJ (version 1.52t) https://imagej.nih.gov/ij/
Living Image® Software (version 4.4) for IVIS® Spectrum https://www.perkinelmer.com/
QuantStudio 6 and 7 Flex Real-Time PCR System Software https://www.thermofisher.com/ch/en/home/global/forms/life-science/quantstudio-6-7-flex-software.html
LI-COR Image Studio™ Lite Software version 5.2 https://www.licor.com/
ZEISS LSM 700 Zen https://www.zeiss.com/
Leica Application Suite X (LAS X) https://www.leica-microsystems.com/de/produkte/mikroskop-software/p/leica-las-x-ls/
Seurat software v4.0 https://satijalab.org/seurat/
Cell Ranger v5.0.1 https://support.10xgenomics.com/single-cell-vdj/software/pipelines/latest/installation
USCS genome browser via the breast cancer epigenomics track (BC) Hub (https://bchub.epfl.ch)
Scvelo version 0.2.4 https://scvelo.readthedocs.io/
Escape version 1.6.0 www.github.com/ncborcherding/escape
BD FACSDiva Software version 9.0 https://www.bdbiosciences.com/en-ch/products/software/instrument-software/bd-facsdiva-software

For manuscripts utilizing custom algorithms or software that are central to the research but not yet described in published literature, software must be made available to editors and reviewers. We strongly encourage code deposition in a community repository (e.g. GitHub). See the Nature Research guidelines for submitting code & software for further information.

Data

Policy information about availability of data

All manuscripts must include a data availability statement. This statement should provide the following information, where applicable:

- Accession codes, unique identifiers, or web links for publicly available datasets
- A list of figures that have associated raw data
- A description of any restrictions on data availability

The raw and processed scRNA-seq generated in this study have been deposited in the Gene Expression Omnibus (GEO) database under accession code GSE196936 (https://www.ncbi.nlm.nih.gov/geo/query/acc.cgi?acc=GSE196936). Raw data for all figures and supplementary figures are provided with this paper in the Source Data File.

Field-specific reporting

Please select the one below that is the best fit for your research. If you are not sure, read the appropriate sections before making your selection.

☒ Life sciences ☐ Behavioural & social sciences ☐ Ecological, evolutionary & environmental sciences

For a reference copy of the document with all sections, see nature.com/documents/nr-reporting-summary-flat.pdf

Life sciences study design

All studies must disclose on these points even when the disclosure is negative.

Sample size	Sample size was estimated based on previous experience with the experimental approaches (Sflomos, G. et al., Cancer Cell, 2016). For growth analyses, at least 4 mice with at least 2 tumors/mouse are required (total of 8 tumors). For metastasis analyses, at least 5 mice are required.
Data exclusions	For the scRNAseq, cells with less than 500 genes and/or higher than 20% mitochondrial reads were removed from the analysis. For qPCR, replicates in which the CT value is higher than 35 (not detected) were excluded. For growth and metastasis analysis, outliers were removed by calculating the the upper and lower boundary, any value that is 1.5 x Interquartile (IQR) greater than the third quartile was designated as an outlier, and any value that is 1.5 x IQR less than the first quartile was also designated as an outlier. No data were excluded from Immunofluorescence and stereoscope analysis, mammary gland weight, mouse body weight, proliferation analysis, western blot, and collagen quantification.
Replication	All experiments were repeated at least 3 independent times at the exception of the Western blot in Extended Data Figure 7h and 7i, which was used to validate successful overexpression. Replication was successful.
Randomization	Before initiation of treatment (DOX), we ensured that control and treatment cohorts have similar primary tumor radiance values. For the rest of the experiments, no randomization was needed.
Blinding	Image analysis in Figure 2g-n, 4g,m, Figure 3b-d, and Figure 6k-o was carried out by "blinded" experimenter i.e. the experimenter did not know the corresponding genotype. For the rest of the experiments, investigators were not blinded to group allocation. For growth assays and metastasis analysis, no blinding was applied as mice are separated in different cages and labeled with their respective condition. For the rest of the experiments, blinding was not necessary because the readout was automated i.e. qPCR, or run through R i.e. scRNAseq, velocity analysis and the experimenter needs to know the corresponding genotypes/conditions.

Reporting for specific materials, systems and methods

We require information from authors about some types of materials, experimental systems and methods used in many studies. Here, indicate whether each material, system or method listed is relevant to your study. If you are not sure if a list item applies to your research, read the appropriate section before selecting a response.

Materials & experimental systems

n/a	Involved in the study	
☐	☒	Antibodies
☐	☒	Eukaryotic cell lines
☒	☐	Palaeontology and archaeology
☐	☒	Animals and other organisms
☐	☒	Human research participants
☒	☐	Clinical data
☒	☐	Dual use research of concern

Methods

n/a	Involved in the study	
☒	☐	ChIP-seq
☐	☒	Flow cytometry
☒	☐	MRI-based neuroimaging

Antibodies

Antibodies used	CK8 BioLegend MMS-162P-250 1E8<br>DsRed MBL Int. PM005 N/A<br>E-cadherin Cell Signaling 3195S 24E10<br>ER Ventana Medical Systems 790-4324 SP-1<br>GFP Santa Cruz sc-9996 B-2<br>Ki67 ThermoFisher Scientific MA5-14520 SP-6<br>Ki67 ORIGENE TA801577 N/A<br>Lamin B1 Abcam AB16048 N/A<br>Vimentin D21H3 XP Cell Sginaling 5741S N/A<br>p120 BD Biosciences 610133 N/A<br>b-catenin BD Biosciences 610154 N/A<br>Cleaved Cytokeratin-18 Roche 12140322001 M30<br>pHH3 (ser10) Millipore 06-570 3H10<br>p27 Kip1 Cell Signaling 3686 D69C12<br>Zeb1 Novus Biologicals NBP1-05987 N/A<br>Mouse Alexa 488 ThermoFisher Scientific A-11029 N/A<br>Mouse Alexa 568 ThermoFisher Scientific A-10037 N/A<br>Mouse Alexa 647 ThermoFisher Scientific A-31571 N/A<br>Rabbit Alexa 488 ThermoFisher Scientific A-21206 N/A<br>Rabbit Alexa 568 ThermoFisher Scientific A-10042 N/A<br>Rabbit Alexa 647 ThermoFisher Scientific A-31573 N/A<br>Rat Alexa 647 ThermoFisher Scientific A-21247 N/A
Validation	ER, Ki67, cleave CK-18, Vimentin, DsRed (RFP), GFP, Lamin B1, pHH3 antibodies have been previously validated by IF or Western blot in the following publications (Sflomos, G. et al. 2016, Cancer Cell; Ataca, D. et al. 2020, Nature Communications; and Sflomos G. et al. 2021, EMBO Molecular Medicine). Zeb1 antibody has been validated by Western blot according the right molecular weight provided by the manufacturer and publications using this antibody Zhang, X. et al. 2018, Cell Death&Disease. p120, b-catenin, p27 antibodies were validated in our study by IF by looking at their correct cellular localization and double checking with the manufacturer (Cell Signaling, and BD Biosciences).

Eukaryotic cell lines

Policy information about cell lines

Cell line source(s)	MCF-7, HCC1806, and BT20 cell lines were purchased from ATCC. T47D cell line (purchased from ATCC) was a kind gift from the laboratory of Prof. Douglas Hanahan. MCF-7 cells harboring ESR1 mutations and their parental cells were obtained from Prof. Simak Ali.
Authentication	None of the cell lines were authenticated
Mycoplasma contamination	MCF-7 and T47D were tested for mycoplasma contamination and were negative by qPCR. BT20 and HCC1806 were not tested for mycoplasma.
Commonly misidentified lines<br>(See ICLAC register)	No commonly misidentified cell lines were used in this study.

Animals and other organisms

Policy information about studies involving animals; ARRIVE guidelines recommended for reporting animal research

Laboratory animals	All mice were maintained and handled according to Swiss guidelines for animal safety with a 12-h-light-12-h-dark cycle, controlled temperature (22 ± 2 degrees Celsius) and humidity (55% ± 10%) , and food and water ad libitum. Experiments were performed in accordance with protocol VD1865.5 approved by the Service de la Consommation et des Affaires Vétérinaires, Canton de Vaud, Switzerland. NOD.Cg-Prkdcscid Il2rgtm1Wjl/SzJ (NSG) and NOD.Cg-Prkdcscid Il2rgtm1Wjl Tg(CAG-EGFP)1Osb/SzJ (NSG-EGFP) mice were purchased from Charles River and The Jackson Laboratory. 8-16-week-old NSG or NSG-EGF female mice were used in this study.
Wild animals	No wild animals were used in this study.
Field-collected samples	No field-collected samples were used in this study.
Ethics oversight	All mice were maintained and handled according to Swiss guidelines for animal safety and experiments were performed in accordance with protocol VD1865.5 approved by Service de la Consommation et des Affaires Vétérinaires, Canton de Vaud, Switzerland.

Note that full information on the approval of the study protocol must also be provided in the manuscript.

Human research participants

Policy information about studies involving human research participants

Population characteristics	Female patients who are diagnosed with estrogen receptor-positive primary breast cancer and who signed the consent form are included in our study. No age, race, class, .. criteria were applied in the recruitment of patients. These patients should not have been treated prior to sample collection (naive tumors). Patients diagnosed with metastatic tumors (distant metastasis, pleural effusion, or ascites) and who signed the consent form are included in our study.
Recruitment	Inclusion criteria: Invasive carcinoma, larger than 1.5 centimeter, without treatment or refractory to treatment, negative viral tests for HIV and Hepatitis B & C. Exclusion Criteria: Carcinoma < 1.5 centimeter, non-invasive, viral tests positive. Oncologists discussed with patients about tissue donation for research, and patients signed the informed consent. No bias in the selection.
Ethics oversight	The Commission cantonale d'éthique de la recherche sur l'être humain (CER-VD 38/15, PB_2016-01185 (38/15)) approved this study and informed consent was obtained from all subjects.

Note that full information on the approval of the study protocol must also be provided in the manuscript.

Flow Cytometry

Plots

Confirm that:

☒ The axis labels state the marker and fluorochrome used (e.g. CD4-FITC).

☒ The axis scales are clearly visible. Include numbers along axes only for bottom left plot of group (a 'group' is an analysis of identical markers).

☒ All plots are contour plots with outliers or pseudocolor plots.

☒ A numerical value for number of cells or percentage (with statistics) is provided.

Methodology

Sample preparation	Intraductal xenografts of MCF-7:RFP-Luc were grown for 6 months. Mammary glands and lungs from 2 NSG-EGFP mice were collected and were mechanically and enzymatically digested using parallel razor blades and 1.5% Collagenase A/1% hyaluronidase, respectively. Samples were centrifuged at 2,500 RPM at 25°C for 10 minutes, and resuspended in red blood cell lysis buffer (Sigma, R7757) for 3-5 minutes, then diluted in PBS 2% CS, and centrifuged again. Pellet was resuspended in trypsin for 3 minutes at 37°C, then centrifuged again, and resuspended in 0.1 mg/ml DNase (1284932, Roche AG). After centrifugation, pellet was resuspended in 10 ml PBS, 2% CS, filtered through a a 40 um pore size filter (cat#352350, BD Falcon) for FACS.
Instrument	BD FACSAria-II SORP
Software	BD FACSDiva™ Software version 9.0
Cell population abundance	Sorted primary tumor cells were 50,000 cells while lung DTCs were around 1,000 cells.
Gating strategy	GFP-/RFP+/DRAQ7-/Hoechst 33342+ cells were gated and sorted.

☒ Tick this box to confirm that a figure exemplifying the gating strategy is provided in the Supplementary Information.
